# Ablation of BATF Alleviates Transplant Rejection *via* Abrogating the Effector Differentiation and Memory Responses of CD8^+^ T Cells

**DOI:** 10.3389/fimmu.2022.882721

**Published:** 2022-04-19

**Authors:** Shuang Li, Dawei Zou, Wenhao Chen, Yating Cheng, Gavin W. Britz, Yi-Lan Weng, Zhaoqian Liu

**Affiliations:** ^1^ Department of Clinical Pharmacology, Hunan Key Laboratory of Pharmacogenetics, and National Clinical Research Center for Geriatric Disorders, Xiangya Hospital, Central South University, Changsha, China; ^2^ Institute of Clinical Pharmacology, Central South University, Changsha, China; ^3^ Department of Neurosurgery, Houston Methodist Neurological Institute, Houston, TX, United States; ^4^ Center for Neuroregeneration, Houston Methodist Research Institute, Houston, TX, United States; ^5^ Immunobiology & Transplant Science Center, Department of Surgery, Houston Methodist Research Institute & Institute for Academic Medicine, Houston Methodist Hospital, Houston, TX, United States

**Keywords:** BATF, CD8^+^ T cells, effector differentiation, allograft rejection, transplantation, memory

## Abstract

Allogeneic CD8^+^ T cells are prominently involved in allograft rejection, but how their effector differentiation and function are regulated at a transcriptional level is not fully understood. Herein, we identified the basic leucine zipper ATF-like transcription factor (BATF) as a key transcription factor that drives the effector program of allogeneic CD8^+^ T cells. We found that BATF is highly expressed in graft-infiltrating CD8^+^ T cells, and its ablation in CD8^+^ T cells significantly prolonged skin allograft survival in a fully MHC-mismatched transplantation model. To investigate how BATF dictates allogeneic CD8^+^ T cell response, BATF^–/–^ and wild-type (WT) CD8^+^ T cells were mixed in a 1:1 ratio and adoptively transferred into B6.*Rag1* ^–/–^ mice 1 day prior to skin transplantation. Compared with WT CD8^+^ T cells at the peak of rejection response, BATF^–/–^ CD8^+^ T cells displayed a dysfunctional phenotype, evident by their failure to differentiate into CD127^–^KLRG1^+^ terminal effectors, impaired proliferative capacity and production of pro-inflammatory cytokines/cytotoxic molecules, and diminished capacity to infiltrate allografts. In association with the failure of effector differentiation, BATF^–/–^ CD8^+^ T cells largely retained TCF1 expression and expressed significantly low levels of T-bet, TOX, and Ki67. At the memory phase, BATF-deficient CD8^+^ T cells displayed impaired effector differentiation upon allogeneic antigen re-stimulation. Therefore, BATF is a critical transcriptional determinant that governs the terminal differentiation and memory responses of allogeneic CD8^+^ T cells in the transplantation setting. Targeting BATF in CD8^+^ T cells may be an attractive therapeutic approach to promote transplant acceptance.

## Introduction

Alloreactive CD8^+^ T cells play an essential role in transplant rejection (1). In the clinical settings, recent studies have found that the increase of some CD8^+^ T cell effector subpopulations in peripheral is positively correlated with higher allograft rejection rates and worse transplant outcomes (2–4). In the laboratory settings, WT B6 recipients reject the minor histocompatibility antigen-mismatched heart allografts, but CD8^+^ T cell-deficient recipients fail to do so (5). Moreover, we and others have demonstrated previously that lymphopenic mice reconstituted with CD8^+^ T cells alone acutely reject MHC fully mismatched allografts (6, 7), suggesting that the adoptively transferred CD8^+^ T cells differentiate into cytotoxic effector T cells and sequentially drive the rejection of allografts in these lymphopenic recipients (7).

The proper differentiation of alloreactive CD8^+^ T cells into functional effectors is essential for allograft rejection. Following alloantigen stimulation, T cell receptor (TCR) signals trigger allogeneic CD8^+^ T cell activation, proliferation, and differentiation into cytotoxic effector T cells (8, 9). The cytotoxic effector T cells infiltrate allografts, produce cytotoxic molecules and pro-inflammatory cytokines, and cause allograft damage (4). We have demonstrated previously that low transplant antigen load induces effector differentiation of anti-graft CD8^+^ T cells and transplant rejection, whereas high transplant antigen load promotes the exhaustive differentiation of CD8^+^ T cells and transplant acceptance in a male-to-female skin transplantation model (10). To prevent transplant rejection and promote transplant acceptance, it is critical to understand the basic molecular mechanisms underlying the effector differentiation of CD8^+^ T cells.

The differentiation of CD8^+^ T cells into cytotoxic effector T cells is delicately controlled by some transcriptional networks. For instance, transcription factors IRF4 (11, 12), T-bet (13), ID2 (14), and ZEB2 (13, 15) promote effector T cell differentiation, whereas TCF1 (16), ID3 (17, 18), and BACH2 (19) restrain effector T cell differentiation and function. Of note, our recent works have demonstrated an essential role for IRF4, a pioneer factor induced by TCR signals (20), in terminal effector T cell differentiation and transplant rejection (7, 21). However, the transcriptional programs that control the alloreactive CD8^+^ T cell responses remain poorly defined.

BATF belongs to the activator protein-1 (AP-1) family, and it is expressed predominantly in hematopoietic cells (22, 23). BATF is induced upon T cell activation and it plays an essential role in T cell differentiation and function (24). In CD4^+^ T cells, BATF is critical for T helper (Th) cell lineage commitment, including Th2 (25), Th9 (26), Th17 (27), and Tfh (28) cell differentiation. In CD8^+^ T cells, BATF has been shown to promote and sustain effector T cell differentiation and function against cancers (29, 30) and infections (24, 31, 32). However, the role of BATF in allogeneic CD8^+^ T cell responses remains unclear.

We have found previously that a single MHC class II-mismatched B6-bm12 heart allografts did not develop chronic cardiac allograft vasculopathy and were accepted by BATF^–/–^ recipients (33). Herein, we investigated the role of BATF in allogeneic CD8^+^ T cell responses. B6.*Rag1* ^–/–^ mice were reconstituted with either WT or BATF^–/–^ naïve CD8^+^ T cells, followed by Balb/c skin transplantation. We found that BATF deficiency in CD8^+^ T cells significantly prolonged the skin allograft survival upon adoptive transfer into lymphopenic mice. Moreover, BATF-deficient CD8^+^ T cells displayed lower frequencies in peripherals, barely infiltrated skin allografts, lost cytokine and cytotoxic molecule production, and failed to differentiate into CD127^–^KLRG1^+^ terminal effectors. Mechanistically, BATF deficiency in CD8^+^ T cells may perturb transcriptional networks that control the effector programs of CD8^+^ T cells. At the memory phase, BATF deficiency in CD8^+^ T cells impaired their memory responses against donor-type skin allografts. Hence, BATF in CD8^+^ T cells might serve as a potential target to develop new therapeutic approaches to prevent allograft rejection.

## Materials and Methods

### Mice

C57BL/6 (B6), B6.SJL CD45.1 congenic, B6.*Rag1* ^–/–^, Balb/c, and BATF^–/–^ mice were purchased from the Jackson Laboratory (Bar Harbor, MA), and were housed in a specific pathogen-free facility at Houston Methodist Research Institute in Houston, Texas. Six- to Ten- week-old mice were randomly assigned to all the experiments. All animal-related experiments were approved by the Houston Methodist Animal Care Committee in accordance with the institutional animal care and use guidelines with IACUC protocol number IS00005481.

### Reconstitution of B6.*Rag1* ^–/–^ Mice With Naïve CD8^+^ T Cells

As previously described (7), CD8^+^ T cells were isolated from the spleens and lymph nodes of WT or BATF^–/–^ mice using the Dynabeads untouched mouse CD8 cells kit (Thermo Fisher Scientific). To obtain CD44^low^ naïve CD8^+^ T cells, the Depletion Dynabeads (Thermo Fisher Scientific) and Anti-CD44 mAb (clone IM7, Biolegend) were used to further purify the isolated CD8^+^ T cells. The purity of the isolated CD8^+^ T cells was around 95% prior to cell transfer. B6.*Rag1* ^–/–^ mice were adoptively transferred with either 1 x 10^6^ WT naïve CD8^+^ T cells or 1 x 10^6^ BATF^–/–^ naïve CD8^+^ T cells 1 day prior to skin transplantation.

### Adoptive Co-Transfer of CD8^+^ T Cells

In the co-transfer experiments, 0.5 x 10^6^ WT naïve CD8^+^ T cells and 0.5 x 10^6^ naïve BATF^–/–^ CD8^+^ T cells were mixed in a 1:1 ratio and adoptively transferred into B6.*Rag1* ^–/–^ mice *via* tail veins. These mice were transplanted with Balb/c skin allografts 1 day later. On day 11 post skin transplantation, the phenotypes of the adoptively transferred CD8^+^ T cells were determined by an LSR II or Fortessa flow cytometer (BD Biosciences).

### Murine Skin Transplantation

As previously described (7), ~1.0 x 1.0 cm skin allografts from Balb/c mice were transplanted onto the backs of either WT B6 or B6.*Rag1* ^–/–^ recipients. A gauze pad and a secure doubled-up bandage were used to protect the transplanted skins from irritating agents until day 7 post skin transplantation. On day 7 post-skin grafting, the sutures, gauzes, and bandages were removed by sterile scissors. Skin graft survival was monitored daily until 50 days post skin grafting. Allograft rejection was considered when necrosis of the donor skin tissue was > 90%.

### Isolation of Skin Allograft-Infiltrating Cells

Skin allografts were harvested, cut into small pieces, and incubated in a solution containing 450 U/ml collagenase I (Thermo Fisher Scientific) and 60 U/ml DNase-I (Thermo Fisher Scientific) in Dulbecco’s Modification of Eagle’s Medium (DMEM, Corning) at 37°C for 45 min. After the incubation, the cells were pressed through a 40 μm filter, and further purified using the 44% Percoll (Cytiva) gradient by centrifugation. The isolated cells were counted, stained with fluorescence-conjugated antibodies, and analyzed by an LSR II or Fortessa flow cytometer.

### T Cell Proliferation Assay

WT and BATF^–/–^ naïve CD8^+^ T cells (total 1 x 10^6^ cells) were mixed in a 1:1 ratio, stained with CellTrace Violet (CTV, Thermo Fisher Scientific) according to the manufacturer’s protocol, and adoptively transferred into B6.*Rag1* ^–/–^ mice that received Balb/c skins 1 day later. On day 7 post skin grafting, the CTV fluorescence of CD8^+^ T cells in spleens and draining lymph nodes (dLNs) was determined by an LSR II or Fortessa flow cytometer.

### Flow Cytometric Analysis

Fluorochrome-conjugated antibodies specific for mouse CD8 (clone 53-6.7), CD3 (17A2), CD45 (30-F11), CD45.2 (104), CD62L (MEL-14), CD44 (IM7), CD45.1 (A20), CD127 (A7R34), CX3CR1 (SA011F11), granzyme A (3G8.5), granzyme B (QA16A02), Ki67 (16A8), KLRG1 (2F1/KLRG1), IFN-γ (XMG1.2), PD-1 (29F.1A12), T-bet (4B10), TCR-β (H57-597), and TNF-α (MP6-XT22) were purchased from BioLegend. Goat anti-rabbit IgG (Catalog A-21244), purified antibody specific for BATF (D7C5), and fluorochrome-conjugated antibody specific for TCF1 (C63D9) were purchased from Cell Signaling Technology. A fluorochrome-conjugated antibody specific for TOX (REA473) was purchased from Miltenyi Biotec.

Flow cytometric analysis was performed as previously described (21). In brief, cells from spleens, dLNs, and skin allografts were stained with the Zombie Aqua Fixable Viability Kit (BioLegend) first, then incubated with the above antibodies, and analyzed on an LSR II or Fortessa flow cytometer later. To determine the intracellular expression of cytokines and cytotoxic molecules, cells were stimulated with 50 ng/ml phorbol 12-myristate 13-acetate (PMA; Sigma-Aldrich) and 500 ng/ml ionomycin (Sigma-Aldrich) in the presence of GolgiStop (BD Biosciences) for 4 hours, then were determined by Cytofix/Cytoperm solution (BD Biosciences) as previously described (21). The expression of transcription factors was determined by the Foxp3/Transcription Factor Staining Buffer Set (Thermo Fisher Scientific) as previously described (21). Data were processed using the FlowJo v10 software (Tree Star, Inc.).

### Statistical Analysis

Data were represented as mean ± SD and analyzed with Prism version 8 (GraphPad Software). The *p* values of the survival of skin allografts were determined with the Mann-Whitney test. Differences were calculated by the unpaired Student’s *t*-test. *p* < 0.05 was considered as statistically significant.

## Results

### Graft-Infiltrating CD8^+^ T Cells Express High Levels of BATF, and BATF Ablation in CD8^+^ T Cells Prolongs Skin Allograft Survival

**Figure 1:**
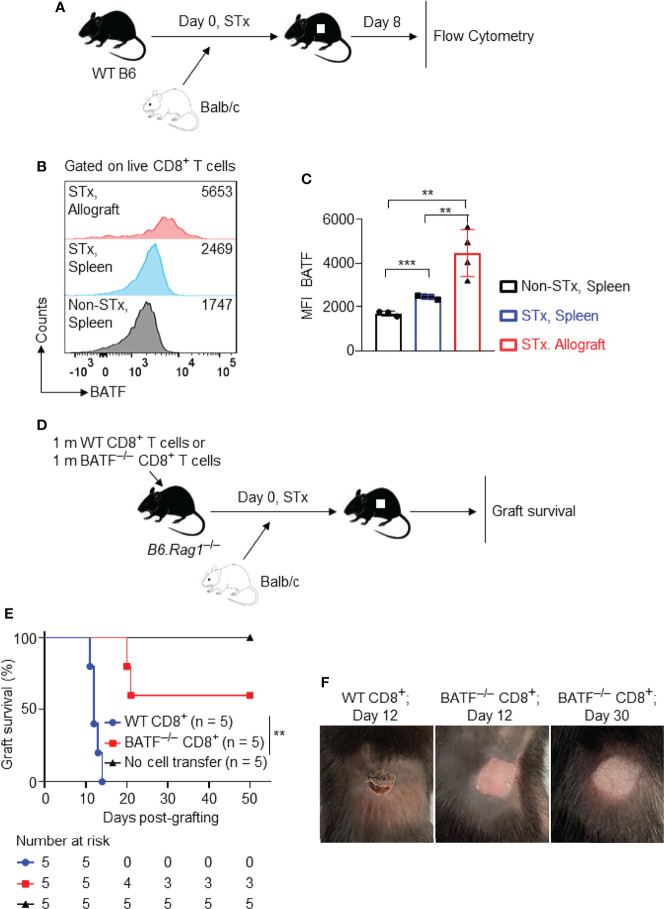
Graft-infiltrating CD8^+^ T cells highly express BATF, and BATF deficiency in CD8^+^ T cells prolongs skin allograft survival. Wild-type B6 recipients were transplanted with Balb/c skin allografts or left untransplanted on day 0. The expression levels of BATF in CD8^+^ T cells in these recipients were detected by flow cytometry on day 8 post skin grafting. All plots were gated on live CD8^+^ T cells. **(A)** Schematic of the experimental design. **(B, C)** Representative plots and bar graphs display the BATF expression levels in CD8^+^ T cells from spleens and skin allografts. Data represent the mean ± SD (n = 3-4). **p < 0.01, ***p < 0.001; (unpaired Student’s *t*-test). B6.*Rag1* ^–/–^ hosts were adoptive transferred with either 1 x 10^6^ WT CD8^+^ T cells or 1 x 10^6^ BATF^–/–^ CD8^+^ T cells, followed by Balb/c skin transplantation. **(D)** Schematic of the experimental design. **(E)** % skin allograft survival (n = 5). **p < 0.01; (Mann-Whitney test). **(F)** Representative images of the skin allografts on B6.*Rag1* ^–/–^ recipients at indicated days post skin grafting.

To investigate the role of BATF in allograft rejection, we first determined its expression levels in allogeneic T cells following transplantation. WT B6 mice were transplanted with Balb/c skin allografts or left untransplanted. On day 8 post skin transplantation, the BATF expression levels in splenic and graft-infiltrating CD8^+^ T cells were determined by flow cytometric analysis (**Figure 1A**). **Supplementary Figure 1** showed the gating strategy to detect the live graft-infiltrating CD8^+^ T cells (**Supplementary Figure 1A**). We found that splenic CD8^+^ T cells from skin grafted recipients expressed a higher level of BATF than those from naïve B6 mice. Of note, compared with splenic CD8^+^ T cells from naïve mice and skin grafted recipients, graft-infiltrating CD8^+^ T cells from skin grafted recipients expressed a significantly higher level of BATF (**Figures 1B, C**). The higher expression levels of BATF were associated with the higher Ki67 and PD-1 expression in graft-infiltrating CD8^+^ T cells (**Supplementary Figures 1B, C**).

To investigate whether BATF deficiency in CD8^+^ T cells affects transplant outcome, B6.*Rag1* ^–/–^ mice were reconstituted with 1 x 10^6^ WT CD8^+^ T cells or 1 x 10^6^ BATF^–/–^ CD8^+^ T cells on day –1, and were transplanted with Balb/c skin allografts on day 0 (**Figure 1D**). B6.*Rag1* ^–/–^ mice do not contain T cells or B cells and did not reject skin allografts without cell transfer (Mean survival time [MST] > 50 days; n = 5). B6.*Rag1* ^–/–^ mice adoptively transferred with WT CD8^+^ T cells acutely rejected skin allografts in 14 days (MST = 12.4 ± 1.14 days; n = 5). In contrast, BATF deficiency in CD8^+^ T cells significantly prolonged skin allograft survival in B6.*Rag1* ^–/–^ recipients (MST = 38.2 ± 16.2 days; n = 5) (**Figure 1E**). **Figure 1F** showed the representative images of the acutely rejected skin allograft in B6.*Rag1* ^–/–^ mice that were transferred with WT CD8^+^ T cells (left image), and the accepted skin allografts in B6.*Rag1* ^–/–^ mice that were reconstituted with BATF^–/–^ CD8^+^ T cells at indicated days post skin transplantation (middle and right images) (**Figure 1F**).

Taken together, BATF is highly expressed in graft-infiltrating CD8^+^ T cells, and BATF deletion in CD8^+^ T cells prolongs skin allograft survival.

### BATF-Deficient CD8^+^ T Cells Display a Defect in Proliferation and Barely Infiltrate Into Skin Allografts

**Figure 2:**
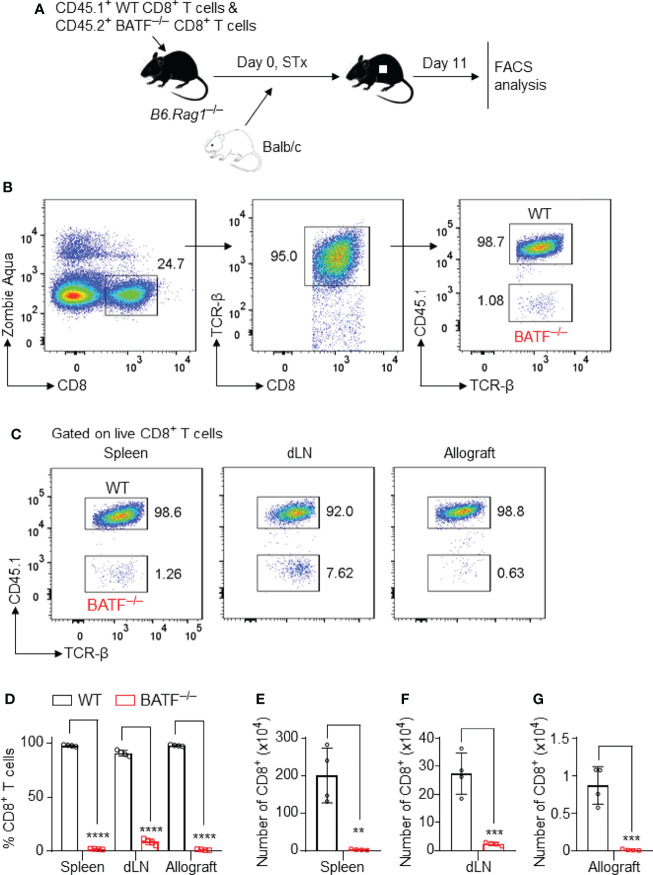
BATF-sufficient CD8^+^ T cells outcompete their BATF-deficient counterparts in infiltration into skin allografts. B6.*Rag1* ^–/–^ recipients were adoptively transferred with a mixture of 0.5 x 10^6^ CD45.1^+^ WT CD8^+^ T cells plus 0.5 x 10^6^ CD45.2^+^ BATF^–/–^ CD8^+^ T cells, followed by Balb/c skin transplantation. The frequencies of the transferred CD8^+^ T cells were determined on day 11 post skin grafting. **(A)** Schematic of the experimental design. **(B)** Gating strategy for detecting live WT and BATF^–/–^ CD8^+^ T cells. **(C, D)** Representative plots and bar graphs show the percentages of the CD45.1^+^ WT and CD45.2^+^ BATF^–/–^ cells among live CD8^+^ T cells. **(E–G)** Bar graphs display the absolute cell numbers of WT and BATF^–/–^ CD8^+^ T cells. Data represent the mean ± SD (n = 4). **p < 0.01, ***p < 0.001, ****p < 0.0001; (unpaired Student’s *t*-test).

To investigate the mechanisms by which BATF deficiency in CD8^+^ T cells impairs their ability to reject skin allografts, we performed a co-transfer experiment, in which CD45.2^+^ BATF^–/–^ CD8^+^ T cells and CD45.1^+^ WT CD8^+^ T cells (with or without CTV labeling) were mixed in a 1:1 ratio, and were adoptively transferred into syngeneic B6.*Rag1* ^–/–^ hosts. Balb/c tail skin allografts were transplanted onto these host mice 1 day later. The cell states of the transferred T cells were analyzed on days 7 and 11 post skin transplantation (**Figure 2A**).

The gating strategies were used to identify the adoptively transferred CD45.2^+^ BATF^–/–^ and CD45.1^+^ WT CD8^+^ T cells (**Figure 2B** and **Supplementary Figure 2A**). In this co-transfer setting, the majority of WT CD8^+^ T cells completely lost CTV fluorescence, whereas ~60% of the BATF^–/–^ CD8^+^ T cells did not completely lose CTV fluorescence in both spleens and dLNs, indicating the impaired proliferation of BATF^–/–^ CD8^+^ T cells (**Supplementary Figure 2A**). The percentages of the transferred BATF^–/–^ CD8^+^ T cells were significantly lower than their WT counterparts in spleens and dLNs. Importantly, the out-competition of BATF^–/–^ CD8^+^ T cells by their WT counterparts was shown in skin allografts. The percentage of the transferred WT CD8^+^ T cells in the skin allografts was approximately 99%, whereas the transferred BATF^–/–^ CD8^+^ T cells only accounted for 1% (**Figures 2C, D**). In line with the low frequencies, BATF^–/–^ CD8^+^ T cells exhibited significantly lower absolute cell numbers in the spleens, dLNs, and allografts compared to WT CD8^+^ T cells (**Figures 2E**–**G**). Hence, our results showed that BATF deficiency in CD8^+^ T cells impairs their infiltration into skin allografts.

### BATF-Deficient CD8^+^ T Cells Fail to Differentiate Into Terminal Effector Cells in Response to Alloantigen Stimulation

**Figure 3:**
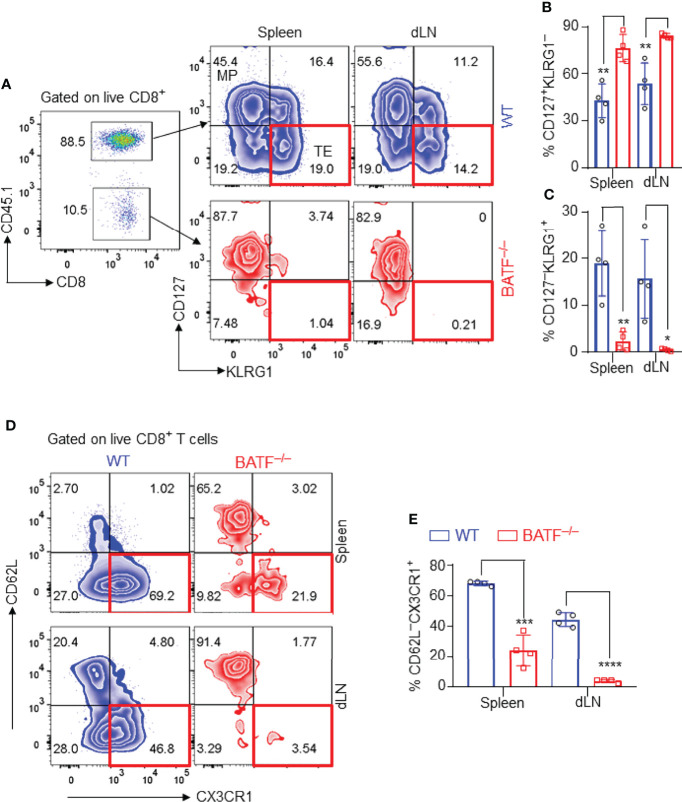
BATF-deficient CD8^+^ T cells fail to differentiate into terminal effector cells in recipients. The effector phenotypes of the transferred CD8^+^ T cells in B6.*Rag1* ^–/–^ recipients were analyzed on day 11 post skin transplantation. **(A–C)** Representative plots and bar graphs show % CD127^–^KLRG1^+^ terminal effector cells and % CD127^+^KLRG1^–^ cells. **(D, E)** Representative plots and bar graphs show % CD62L^–^CX3CR1^+^ effector cells. Data represent the mean ± SD (n = 4). *p < 0.05, **p < 0.01, ***p < 0.001, ****p < 0.0001; (unpaired Student’s *t*-test).

We next identified the effector phenotypes of the transferred CD8^+^ T cells in B6*.Rag1* ^–/–^ recipients at day 11 post skin transplantation. In response to alloantigen stimulation, a great proportion of WT CD8^+^ T cells differentiated into CD127^–^KLRG1^+^ terminal effector cells, while most BATF-deficient CD8^+^ T cells maintained a CD127^+^KLRG1^–^ memory precursor phenotype in both spleens and dLNs (**Figures 3A**–**C**). CX3CR1 is an important chemokine receptor that is associated with the degree of CD8^+^ T cell effector differentiation (34). We found that most WT CD8^+^ T cells expressed high levels of CX3CR1 and differentiated into CD62L^–^CX3CR1^+^ effector cells in spleens and dLNs. In contrast, BATF^–/–^ CD8^+^ T cells did not upregulate CX3CR1 expression and largely retained CD62L^+^CX3CR1^–^ naïve-like phenotypes (**Figures 3D, E**). Taken together, BATF deficiency in CD8^+^ T cells abrogates their terminal effector differentiation in response to alloantigen stimulation.

### BATF Deficiency in CD8^+^ T Cells Diminishes the Production of Effector Cytokines and Cytotoxic Molecules in Lymphopenic Recipients

**Figure 4:**
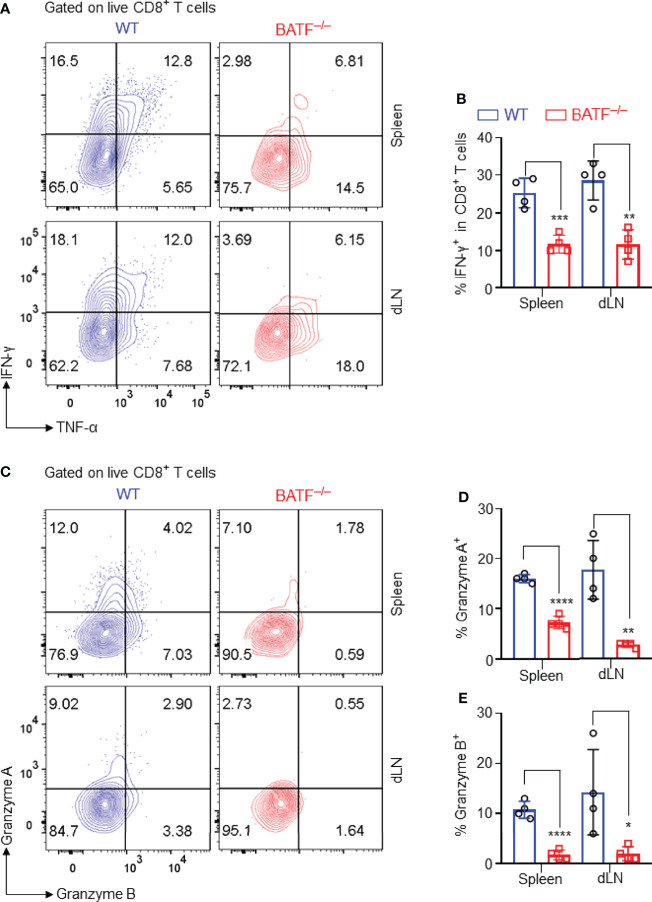
BATF deficiency in CD8^+^ T cells impairs their expression of proinflammatory cytokines and cytotoxic molecules. The production of the effector cytokines and cytotoxic molecules of the adoptively transferred CD8^+^ T cells in recipients were analyzed on day 11 post skin grafting. All plots were gated on live CD8^+^ T cells. **(A, B)** Representative plots and bar graph display % IFN-γ^+^ cells in spleens and dLNs. **(C–E)** Representative plots and bar graphs display % Granzyme A^+^ cells and % Granzyme B^+^ cells. Data represent the mean ± SD (n = 4). *p < 0.05, **p < 0.01, ***p < 0.001, ****p < 0.0001; (unpaired Student’s *t*-test).

The production of pro-inflammatory cytokines and cytotoxic molecules is one of the effector features of CD8^+^ T cells. We thus analyzed the production of effector cytokines and cytotoxic molecules of the adoptively transferred CD8^+^ T cells in B6.*Rag1* ^–/–^ recipients. We found that BATF^–/–^ CD8^+^ T cells expressed significantly lower levels of IFN-γ than did WT counterparts in spleens and dLNs (**Figures 4A, B**). Upon recognition of allograft parenchymal cells, allogeneic effector CD8^+^ T cells produce Granzymes, induce parenchymal cell death, and thus cause graft failure (35). We found that WT CD8^+^ T cells produced significantly higher levels of granzyme A and granzyme B than did BATF^–/–^ CD8^+^ T cells in both spleens and dLNs (**Figures 4C**–**E**). Collectively, BATF deficiency in CD8^+^ T cells impairs their ability to produce proinflammatory cytokines and cytotoxic molecules in lymphopenic recipients.

### BATF Deficiency may Perturb Transcriptional Networks That Control the Effector Program of CD8^+^ T Cells

**Figure 5:**
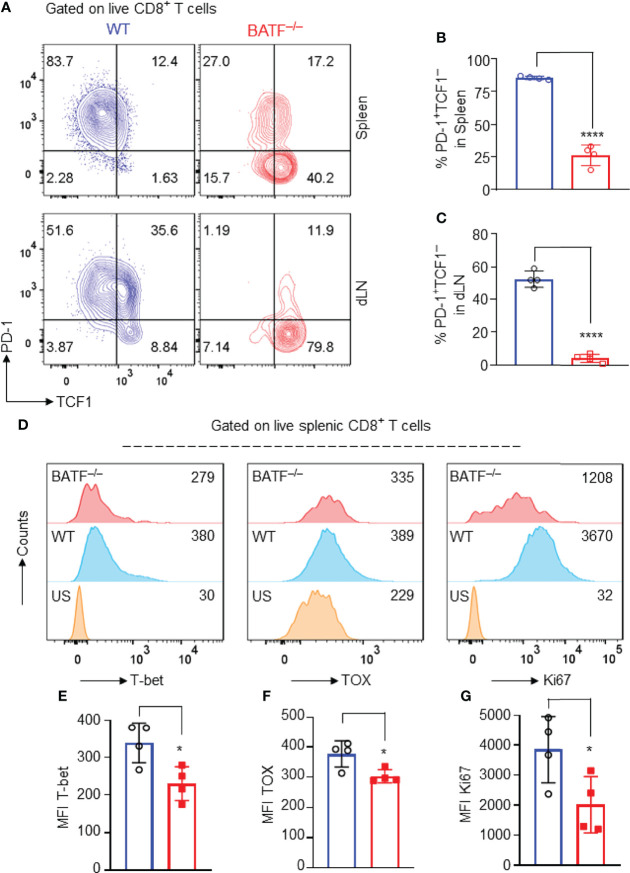
BATF deficiency may perturb the transcriptional networks that control the effector programs of CD8^+^ T cells. B6.*Rag1* ^–/–^ mice were reconstituted with a mixture of WT and BATF^–/–^ CD8^+^ T cells, and were transplanted with Balb/c skins 1 day later. The expression levels of several transcription factors were determined on day 11 after skin transplantation. All plots were gated on live splenic CD8^+^ T cells. **(A–C)** Representative plots and bar graphs show the expression levels of TCF1 and PD-1 of the transferred WT and BATF^–/–^ CD8^+^ T cells. **(D–G)** Representative histograms and bar graphs display the expression levels of T-bet, TOX and Ki67 in the transferred CD8^+^ T cells. Data represent the mean ± SD (n = 4). *p < 0.05, ****p < 0.0001; (unpaired Student’s *t*-test).

Several transcriptional factors have been shown to play key roles in controlling the effector programs of CD8^+^ T cells. Transcriptional factors T-bet (13), TOX (36), and Ki67 (37) are positively involved in the effector programs of CD8^+^ T cells, whereas TCF1 is highly expressed in naïve T cells, directly represses transcription factors involved in effector programs, and restrains their effector function and differentiation (38). In response to allograft simulation, most WT CD8^+^ T cells lost TCF1 expression and upregulated the expression of inhibitory receptor PD-1, displaying a TCF1^–^PD1^+^ effector phenotype. By contrast, BATF^–/–^ CD8^+^ T cells retained high expression levels of TCF1, expressed low levels of inhibitory receptor PD-1, and largely displayed a TCF1^+^PD-1^–^ compromised phenotype (**Figures 5A**–**C**). In addition, the expression levels of T-bet, TOX and Ki67 in BATF-deficient CD8^+^ T cells were significantly lower than those in WT counterparts (**Figures 5D**–**G**). Hence, BATF deficiency may perturb transcriptional networks that control the effector program of CD8^+^ T cells.

### BATF Is Required for Memory Responses Against the Allogeneic Transplant Antigens

**Figure 6:**
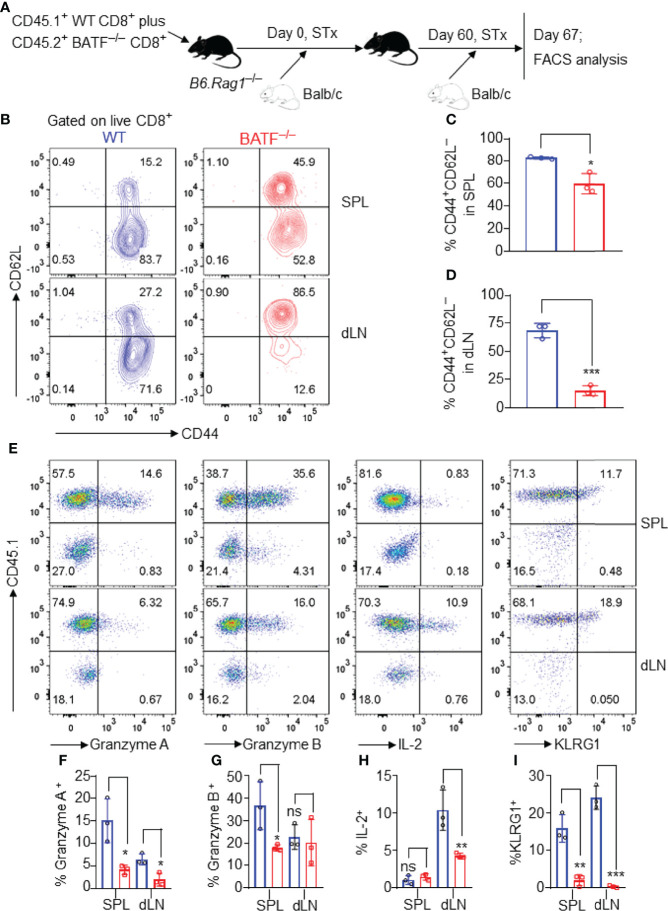
BATF-deficient CD8^+^ T cells display impaired memory responses upon allogeneic antigen re-stimulation. B6.*Rag1* ^–/–^ mice were reconstituted with a mixture of WT and BATF CD8^+^ T cells^–/–^ (in a 1:1 ratio) on day -1, transplanted with Balb/c skins on day 0, and re-transplanted with Balb/c allografts on day 60 post-initial transplantation. On day 7 post-secondary transplantation, the cell states of the transferred CD8^+^ T cells were determined by flow cytometry. All the representative plots are gated on live CD8^+^ T cells. **(A)** Schematic of the experimental design. **(B–D)** Representative plots and bar graphs show % CD44^+^CD62L^–^ cells. **(E–I)** Representative plots and bar graphs display the expression of Granzyme A, Granzyme B, IL-2, and KLRG1. Data represent the mean ± SD (n = 3). ns, *p* > 0.05, *p < 0.05, **p < 0.01, ***p < 0.001; (unpaired Student’s *t*-test).

We next investigated the role of BATF in allogeneic memory responses. A mixture of 0.5 x 10^6^ CD45.2^+^ BATF^–/–^ plus 0.5 x 10^6^ CD45.1^+^ WT CD8^+^ T cells was adoptively transferred into B6.*Rag1* ^–/–^ mice that received Balb/c skins 1 day later. The Balb/c skin allografts were rejected within 16 days post initial transplantation (data not shown). On day 60 post initial transplantation, these mice were re-transplanted with Balb/c skins. The cell states of the CD8^+^ T cells were determined by flow cytometry on day 67 post-initial transplantation (**Figure 6A**).

As shown in **Figures 6B**–**D**, most WT CD8^+^ T cells lost CD62L expression and developed into CD62L^–^CD44^+^ effector memory-like cells. By contrast, BATF^–/–^ CD8^+^ T cells largely maintained CD62L expression, and barely developed into CD62L–CD44^+^ cells in both spleens and dLNs (**Figures 6B**–**D**). Upon allogeneic antigen re-stimulation, WT CD8^+^ T cells mounted a more robust response, evident by the higher expression levels of Granzyme A, Granzyme B, IL-2 and KLRG1, than did BATF-deficient counterparts (**Figures 6E**–**I**). Therefore, BATF deficiency in CD8^+^ T cells impairs their memory response against allogeneic antigen re-stimulation.

## Discussion

T cells are necessary and sufficient to mediate allograft rejection (39). However, the transcriptional regulation of the effector programs in allogeneic CD8^+^ T cells has not been fully elucidated. In the current study, we found that the BATF deletion in CD8^+^ T cells impaired their abilities to reject allografts upon adoptive transfer into immunocompromised recipients. Co-transfer experiments showed that BATF-deficient CD8^+^ T cells displayed a defect in proliferation, lost the production of proinflammatory cytokines and cytotoxic molecules, failed to differentiate into terminal effector cells, and showed an inferior capacity to infiltrate into allografts. Mechanistically, BATF deletion may perturb the interplays between BATF and some key transcription factors that govern the effector differentiation of CD8^+^ T cells. Hence, BATF deficiency in CD8^+^ T cells disrupts their differentiation into terminal effector cells and prolongs transplant survival.

BATF is a crucial transcription factor required for effector differentiation of CD8^+^ T cells. Upon TCR stimulation, T cells upregulate BATF expression and initiate a transcriptional reprogramming that promotes effector differentiation (24, 31). We found that BATF expression was upregulated in splenic and graft-infiltrating CD8^+^ T cells from recipient mice compared with that in splenic CD8^+^ T cells from naïve mice, indicating a role for BATF in reprogramming allogeneic T cell response upon alloantigen stimulation. Indeed, BATF is required at the earliest stages of effector CD8^+^ T cell differentiation (24). Shortly after activation, BATF is able to directly bind to and promote the transcription of lineage-specific genes required for effector differentiation (24). In the setting of infections, the impaired effector differentiation and function of BATF-deficient CD8^+^ T cells eventually results in the defeats to eliminate lymphocytic choriomeningitis virus (LCMV) infections (24, 31, 32). The requirement of BATF family transcription factors in effector differentiation is also demonstrated in other settings (40–42). Consistent with these findings, our data showed that BATF-deficient CD8^+^ T cells exhibited a compromised effector phenotype and lost the capacity to mediate transplant rejection. The inability to acquire effector features of CD8^+^ T cells is likely to be a major determinant of the prolonged allograft survival. Other than the impaired effector differentiation, there are several reasons that may contribute to the prolonged allograft survival. For instance, deletion of BATF in CD8^+^ T cells may impair their homeostatic proliferation and increase their apoptosis upon transfer into lymphopenic hosts. Of interest, BATF is required for the expression of gut-homing receptors of T cells, and BATF-deficient T cells fail to induce colitis (43). In the setting of transplantation, BATF may be also required for the expression of some skin-homing receptors on CD8^+^ T cells. Thus, BATF deletion in CD8^+^ T cells may impair their migration to skin allografts and their capacity to mediate skin allograft rejection.

The interplays between BATF and several key transcription factors coordinately regulate the effector differentiation of CD8^+^ T cells. For example, TCF1 is highly expressed in naïve T cells, maintains their stem-like properties, and restrains their effector differentiation (44). Danilo et al. have demonstrated previously that TCF1 repression *via* IL-12-induced STAT4 activation facilitates the effector differentiation of CD8^+^ T cells (44). A similar role of TCF1 has also been found in anti-viral response. Tiemessen et al. have found previously that TCF1 deficiency promotes the generation of anti-viral effector CD8^+^ T cells, and thus, enhances virus clearance (16). In contrast to the role of TCF1 in restraining effector differentiation, T-bet plays an essential role in promoting effector T cell differentiation, especially in Th1, and Tc1 cell differentiation (45, 46). T-bet-deficient T cells display compromised effector function against infections (13, 47), and tumors (48, 49). TOX and Ki67 expression levels have been shown to reflect an activated and proliferative T cell state (36, 50, 51). Of interest, we found that BATF-deficient CD8^+^ T cells retained TCF1^hi^ expression and did not upregulate T-bet, TOX, and Ki67 expression upon alloantigen stimulation. Hence, BATF deficiency may disturb the coordinate regulation between BATF and other key transcriptional factors that control the effector program in allogeneic CD8^+^ T cells.

BATF regulates T cell effector differentiation *via* epigenetic remodeling, metabolic reprogramming, or direct binding to lineage-specific genes, among which the transcriptional regulation fundamentally determined the expression of lineage-specific genes and sequentially the cell fate decisions (52). Of interest, the binding affinities of BATF to the target genes are significantly impaired in the absence of IRF4, a cooperating binding partner of BATF (53). BATF forms a trimeric complex with Jun family proteins and IRF4, binds to AP1–IRF composite elements, and regulates the transcription of genes related to cell fate decisions (53, 54). Recent studies have found that BATF and IRF4 cooperate to directly bind to and regulate gene expressions in T cells, such as *Tcf7* and *Tbx21* (encoding TCF1 and T-bet, respectively) (24, 55). Of note, we have found previously that the deletion of IRF4 in T cells promotes transplant acceptance (21), and that IRF4-deficient CD8^+^ T cells fail to differentiate into effectors upon transfer into immunocompromised recipients (7). The cooperation between BATF and IRF4 appears to be essential in allogeneic T cell fate decisions.

It is also essential to recognize several limitations in our current study. First, it remained unclear whether the impaired effector differentiation of BATF-deficient CD8^+^ T cells was specific for the allo-reaction. In our study, polyclonal but not allogeneic CD8^+^ T cells were used, and lymphopenia in the immunodeficient hosts may also drive their effector differentiation. However, our results indicated that neither skin transplantation, nor lymphopenia-induced homeostatic proliferation drives the effector differentiation of BATF^–/–^ CD8^+^ T cells. Second, we currently did not include any translational or clinical studies. These studies are important to solidify our findings and will be explored in the future. Third, the mechanism by which BATF controls the CD8^+^ T-cell memory program is complicated and remains largely unclear. How BATF controls the formation, maintenance, and recall response of memory CD8^+^ T cells is important and will be carefully addressed in our future studies.

In summary, we found that BATF-deficient CD8^+^ T cells displayed a compromised effector phenotype and exhibited a suppressed ability to mediate allograft rejection upon adoptive transfer into lymphopenic recipients. Thus, we proposed that targeting BATF in T cells represents an attractive approach to prevent transplant rejection.

## Data Availability Statement

The original contributions presented in the study are included in the article/**Supplementary Material**. Further inquiries can be directed to the corresponding authors.

## Ethics Statement

The animal study was reviewed and approved by Houston Methodist Animal Care Committee.

## Author Contributions

SL, DZ, WC, GB, Y-LW, and ZL conceived and designed the study. SL and DZ collected and assembled data and wrote the manuscript. SL, DZ, and WC analyzed and interpreted experiments. WC and Y-LW provided study materials. GB, Y-LW, and ZL provided administrative support. WC, YC, Y-LW, and ZL revised the manuscript. All authors contributed to the article and approved the final version of the manuscript.

## Funding

This work was supported by grants from Houston Methodist Career Cornerstone Award (to W.C.), NIH (R01ES031511 to Y-LW) and program from Central South University (31801-160170006 to S.L.).

## Conflict of Interest

The authors declare that the research was conducted in the absence of any commercial or financial relationships that could be construed as a potential conflict of interest.

## Publisher’s Note

All claims expressed in this article are solely those of the authors and do not necessarily represent those of their affiliated organizations, or those of the publisher, the editors and the reviewers. Any product that may be evaluated in this article, or claim that may be made by its manufacturer, is not guaranteed or endorsed by the publisher.

## Abbreviations

AP-1, activator protein-1; BATF, basic leucine zipper ATF-like transcription factor; dLN, draining lymph node; LCMV, lymphocytic choriomeningitis virus; MFI, mean fluorescence intensity; MST, mean survival time; PMA, phorbol 12-myristate 13-acetate; STx, skin transplantation; TCR, T cell receptor; Th, T helper; WT, wild-type.
