# Supplementary material for: Ablation of BATF Alleviates Transplant Rejection via Abrogating the Effector Differentiation and Memory Responses of CD8+ T Cells
Source: Front Immunol. 2022 Apr 19;13:882721. doi: 10.3389/fimmu.2022.882721 (PMC9062028; doi:10.3389/fimmu.2022.882721)
Supplement: Supplementary file 1 [file Presentation_1.pptx]

## Slide 1
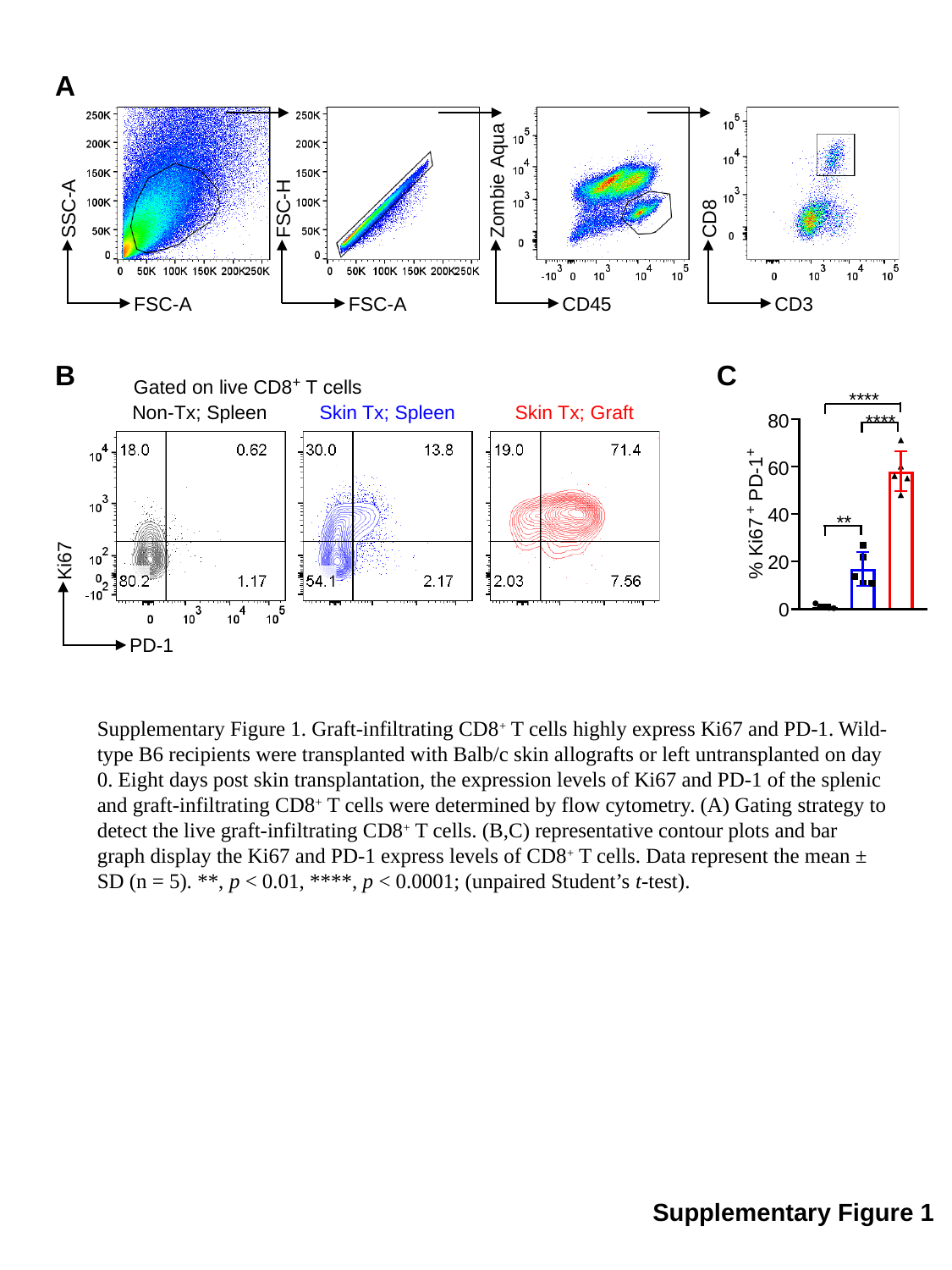

A
Zombie Aqua
CD45
SSC-A
FSC-A
FSC-H
FSC-A
CD8
CD3
B
C
Gated on live CD8+ T cells
Non-Tx; Spleen
Skin Tx; Spleen
Skin Tx; Graft
Ki67
PD-1
****
80
****
60
% Ki67 + PD-1+
40
**
20
0
Supplementary Figure 1. Graft-infiltrating CD8+ T cells highly express Ki67 and PD-1. Wild-type B6 recipients were transplanted with Balb/c skin allografts or left untransplanted on day 0. Eight days post skin transplantation, the expression levels of Ki67 and PD-1 of the splenic and graft-infiltrating CD8+ T cells were determined by flow cytometry. (A) Gating strategy to detect the live graft-infiltrating CD8+ T cells. (B,C) representative contour plots and bar graph display the Ki67 and PD-1 express levels of CD8+ T cells. Data represent the mean ± SD (n = 5). **, p < 0.01, ****, p < 0.0001; (unpaired Student’s t-test).
Supplementary Figure 1

## Slide 2
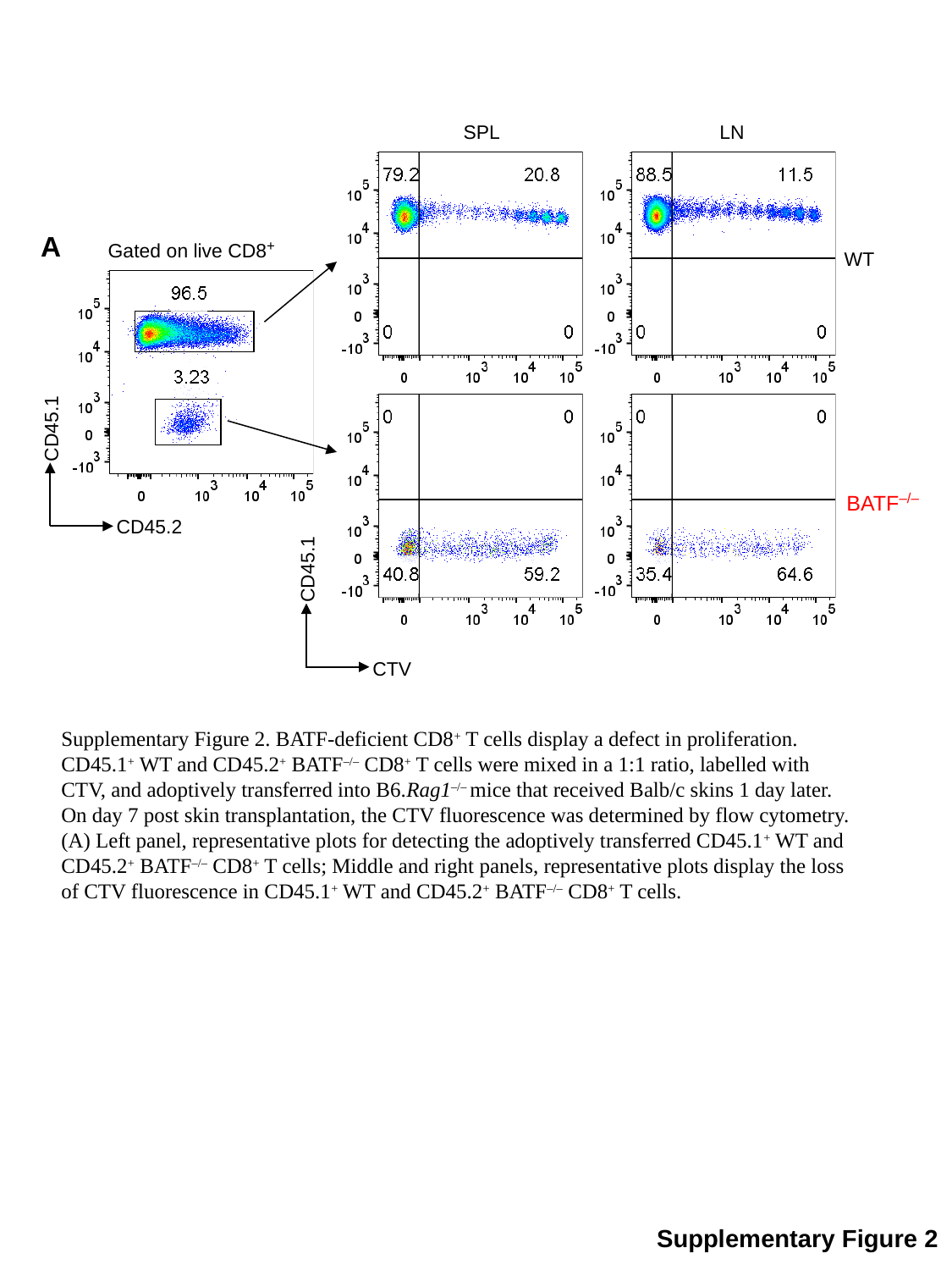

SPL
LN
WT
CD45.1
CD45.2
CD45.1
CTV
BATF–/–
A
Gated on live CD8+
Supplementary Figure 2. BATF-deficient CD8+ T cells display a defect in proliferation. CD45.1+ WT and CD45.2+ BATF–/– CD8+ T cells were mixed in a 1:1 ratio, labelled with CTV, and adoptively transferred into B6.Rag1–/– mice that received Balb/c skins 1 day later. On day 7 post skin transplantation, the CTV fluorescence was determined by flow cytometry.
(A) Left panel, representative plots for detecting the adoptively transferred CD45.1+ WT and CD45.2+ BATF–/– CD8+ T cells; Middle and right panels, representative plots display the loss of CTV fluorescence in CD45.1+ WT and CD45.2+ BATF–/– CD8+ T cells.
Supplementary Figure 2
